# Disease-Modifying Activity of Huperzine A on Alzheimer’s Disease: Evidence from Preclinical Studies on Rodent Models

**DOI:** 10.3390/ijms232315238

**Published:** 2022-12-03

**Authors:** Ye-Piao Yan, Jia-Yue Chen, Jia-Hong Lu

**Affiliations:** State Key Lab of Quality Research in Chinese Medicine, Institute of Chinese Medical Sciences, University of Macau, Macao 999078, China

**Keywords:** Huperzine A, Alzheimer’s disease, animal studies, preclinical studies, meta-analysis, systematic review

## Abstract

(1) Background: Huperzine A, a natural cholinesterase (AChE) inhibitor isolated from the Chinese herb Huperzia Serrata, has been used as a dietary supplement in the United States and a drug in China for therapeutic intervention on Alzheimer’s disease (AD). This review aims to determine whether Huperzine A exerts disease-modifying activity through systematic analysis of preclinical studies on rodent AD models. (2) Methods: Sixteen preclinical studies were included based on specific criteria, and the methodological qualities were analyzed by SYRCLE’s risk of bias tool. Some outcomes were meta-analyzed: latencies and time spent in quadrant of Morris water maze, soluble amyloid-β (Aβ) level measured by ELISA in the cortex and hippocampus, Aβ plaque numbers measured by immunohistochemistry in hippocampus, choline acetyltransferase (ChAT) activity, and AChE activity. Finally, the mechanisms of Huperzine A on AD models were summarized. (3) Conclusions: The outcomes showed that Huperzine A displayed AChE inhibition, ChAT activity enhancement, memory improvement, and Aβ decreasing activity, indicating the disease-modifying effect of Huperzine A. However, due to the uneven methodological quality, the results need to be rationally viewed, and extensively repeated.

## 1. Introduction

Alzheimer’s disease (AD) is the most common neurodegenerative diseases affecting memory and progressive function. AD is characterized by the presence of Aβ plaques and neurofibrillary tangles (NFTs) in the affected region of brain [1]. The prevalence of AD in people over 65 is around 10%. UN Aging Program predicted that the number of elders (over 65 years old) in the world will likely increase from 420 million in 2000 to 1 billion by 2030 [2]. AD has become a great burden for families and society, while the current therapies for AD are unsatisfied. There is an urgent expectation in drug development on AD.

Currently, the most widely accepted hypotheses of AD are the cholinergic hypothesis, the amyloid hypothesis, and tau hypothesis [3]. The cholinergic system is engaged in attention, learning, and memory activities. Cholinergic degeneration was observed in AD, which leads to a cognitive deficit. Cholinergic synapse losses are reported to be affected by Aβ oligomers’ neurotoxicity [4]. Based on cholinergic hypothesis, three cholinesterase inhibitors and an (N-methyl-D-aspartic acid) NMDA receptor antagonist have been applied in the clinical area: donepezil, rivastigmine, galantamine, and memantine.

Aβ is generated from enzymatic cleavages of amyloid precursor protein (APP) by β-secretase and γ-secretase. Aβ is prone to self-aggregating and develop plaques in the AD patients’ brains [5]. Studies reveal that soluble Aβ oligomer levels have stronger correlation to the degree of synaptic loss and cognitive impairment than amyloid plaques [6]. The potential toxicity of Aβ [7] may include over-exciting NMDA receptors [8], forming membrane pores that allow abnormal flow of ions [9], inhibiting Wnt signaling to cause tau hyperphosphorylation [10], and inhibiting proteasome pathway [11].

Highly phosphorylated microtubule-associated protein (MAP) tau is the major component of NFTs and paired helical filaments (PHFs) [12]. Tau has a microtubule-binding domain involved in stabilize tubulin assembly. Hyperphosphorylated tau causes tau mislocalization (from axon into the somatodendritic compartment) and leads to synaptic dysfunction [13]. In recent years, it has been shown that tau spreads from cell to cell like a prion and that tau oligomers will induce the orderly assembly of monomeric tau, which will then spread to other areas of the brain [14]. Tau activates microglia, triggers neuroinflammation, and spreads itself through exosome secretion, which may also be important reasons for the progression of tau pathology [15,16].

Huperzine A was found in the Chinese herb Huperzia Serrata by Chinese scientists in 1980s, showing reversible specific inhibition on acetylcholinesterase [17]. Huperzine A was approved by FDA as a dietary ingredient, while in China it had been approved for the treatment of mild cognitive impairment in various doses and forms. Apart from acetylcholinesterase inhibition, studies reported that Huperzine A attenuated mitochondrial dysfunction induced by Aβ [18] or by ischemia [19], prevented free radical-induced damages [20], antagonized NMDA receptor [21], and stimulated nerve growth factor generation to nourish neurons [22]. Though Huperzine A has been widely used as food supplement or drug to control the symptoms of AD, the disease-modifying properties of Huperzine A has not been well addressed. In this systematic review, the effects and mechanisms of Huperzine A on different rodent AD models are measured and summarized. The bias of included studies are assessed scientifically. The results provide an unbiased analysis of neuroprotective property of Huperzine A as a disease-modifying drug for AD.

## 2. Results

### 2.1. Study Selection

**Figure 1:**
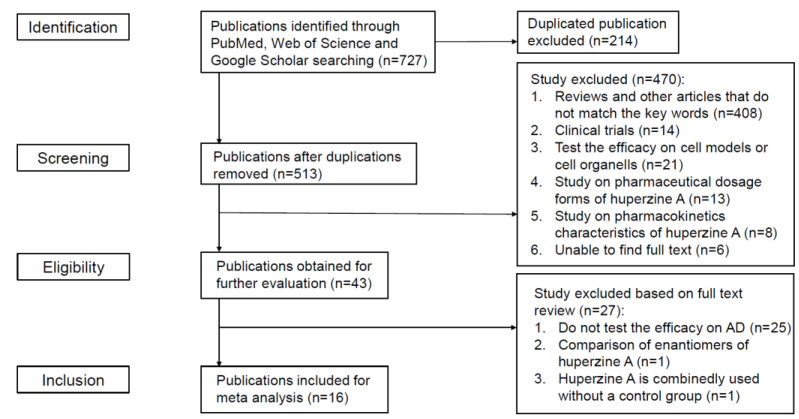
Selection of articles.

At first, we collected 727 articles in total from PubMed, Web of Science, and Google Scholar. Through screening the titles and abstracts, we excluded 711 articles for different reasons that are illustrated in Figure 1. To manage the included articles, we listed their information in Table 1.

### 2.2. Study Characteristics

#### 2.2.1. Animal Models

There are two animal species in our selected 16 articles: rat (n = 8) and mouse (n = 8). Ten of the articles used toxins induced models, including Aβ (n = 4), AF64A (n = 1), AMPA (n = 1), quinolinic acid (n = 1), D-galactose (n = 1), and scopolamine (n = 1). Six of the articles used transgenic mice models, including APPswe/PS1dE9 transgenic mice (n = 5) and senescence-accelerated mouse prone/8 (SAMP8) mice (n = 1). 

Four cholinergic injury models use AF64A, AMPA, quinolinic acid, and scopolamine, respectively. AF64A is known as ethylcholine mustard aziridinium ion [39] and is reported as a cholinergic neurotoxin. Scopolamine is a competitive antagonist of the muscarinic acetylcholine receptor. AMPA and quinolinic acid are excitatory amino acids, which lead to the reduction of cholinergic neurons and the decreased activity of choline acetyltransferase (ChAT). Aβ injection models and APPswe/PS1dE9 transgenic mice simulate the Aβ deposition in brains and can reflect the role of the Aβ pathological pathway in AD. The SAMP8 model is a naturally occurring accelerated aging model which displays learning and memory impairment. The mechanisms and characteristics of these models are summarized in Table 2.

#### 2.2.2. Behavioral Test Analysis

The Morris water maze test is widely used in assessing the drug’s efficacy in AD models as it reflects the spatial memory ability effectively. The training is conducted in a round water pool with a platform hiding under opaque water. The animal is put into the pool and is guided to the platform. After days of training, the animal is allowed to swim freely for 60 s, which is called the probe test. The time spent in the target quadrant and the escape latency will be recorded as the indicators for the spatial memory.

Seven of the sixteen articles carried the test, and two indicators mentioned above were analyzed by the forest plot. We used image J (Rawak Software Inc., Stuttgart, Germany) and RevMan 5.4 (Cochrane Community, London, UK) to quantify and analyze the data. A total of 91 rats and 30 mice were included in meta-analysis of escape latency. Of these, 70 rats and 30 mice were included in the analysis of time spent in the target quadrant. We denoted 1 and 2 for the two doses (0.1 mg/kg and 0.2 mg/kg) used in Wang R. 2001 [34].

**Figure 2:**
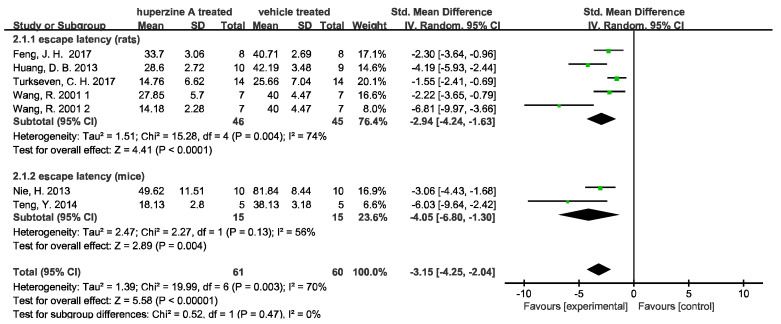
Forest plot for Huperzine A versus vehicle treatment. Outcome: escape latency [24,25,29,31,32,34]. The green squares represent the weight each study contributes to. Line lengths represent 95% confidence intervals for value of each study. Diamonds represent the aggregated results of the meta-analysis. The same below.

**Figure 3:**
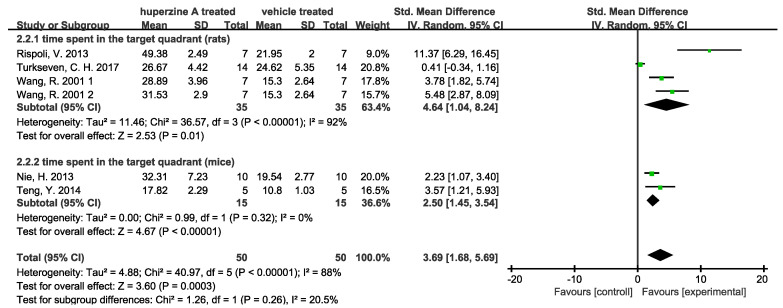
Forest plot for Huperzine A versus vehicle treatment. Outcome: time spent in the target quadrant [29,30,31,32,34].

As Figure 2 shows, Huperzine A treated rats and mice both had significantly reduced escape latencies. However, the heterogeneity of studies is high (I^2^(rats) = 74%, *p* = 0.004; I^2^(mice) = 56%, *p* = 0.13). Similarly, high heterogeneity is observed in time spent in the target quadrant in rat subgroup (I^2^(rats) = 92%) while the increase in time in the mice group is significantly (*p* = 0.004) with low heterogeneity (I^2^ = 0). Such high heterogeneity can be attributed to various AD models used in the studies, including quinolinic acid induced AD, AMPA induced AD, Aβ induced AD, D-galactose induced AD, and APPswe/PS1dE9 mice. Notably, one study [32] indicated that Huperzine A did not increase the time spent in the target quadrant. When this article was excluded, the heterogeneity decreased from 74% to 69%in Figure 2 and from 92% to 74% in Figure 3.

#### 2.2.3. Neuroprotective Mechanisms Analysis

Several neuroprotective mechanisms have been revealed in these studies, including inhibition of Aβ aggregation or related pathways, regulating cholinergic system, attenuating mitochondrial dysfunction, and regulating apoptotic factors as well as anti-oxidation and anti-inflammatory activity.

(1) Inhibition of Aβ pathway

Nine studies showed that Huperzine A has an inhibitory activity on Aβ aggregation or related pathways. Among those studies, three used both ELISA and immunohistochemistry to quantify Aβ level. Wang C. Y. et al. [33] reported the dose dependent decrease in Aβ plaques in the cortex and hippocampus and drastically reduced soluble Aβ40 and Aβ42 by Huperzine A treatment. The APP695 protein level, PS1 level, BACE1 level, and sAPPβ level decreased while sAPPα increased, which indicated the enhancement of nonamyloidogenic pathway in the APP/PS1 mouse brain. Huang X. T. et al. [26] reported the insoluble and soluble Aβ42/Aβ40, the deposition of amyloid plaques, oligomeric Aβ, APP695, and hyperphosphorylated tau levels were reduced by Huperzine A as well. However, they found those changes could be reversed by high iron diet. Xiao X. et al. [37] reported oligomeric Aβ42, Aβ plaques and ABAD level dramatically decreased and mitochondrial dysfunction was attenuated by Huperzine A.

In the remaining six articles, three articles used immunohistochemistry, one used ELISA, and the other two used Thioflavin S staining and Congo red staining to determine the Aβ load. Huang Z. S et al. [27] evaluated the proportion of Aβ positive neurons in cortex and hippocampus and found Huperzine A treatment reduced the number of positive neurons by about 20% compared to SAMP8 vehicle treatment. The expression of BACE and APP mRNA also greatly decreased. Liang Y. Q. et al. [28] injected Aβ peptide into nucleus basalis magnocellularis of rats to model AD and amyloid immunoreactivity indicated the enhancement of Aβ clearance by Huperzine A treatment. Turkseven C. H. et al. [32] reported the reduction of Aβ expression in the cortex of rats which experienced long-term administration of D-galactose after forming menopause. Teng Y. [31] et al. reported Huperzine A reduced Aβ42 level in hippocampus and cortex of Aβ (25–35) injected mice, measured by ELISA. Wang Y. et al. [36] reported the decrease in thioflavin S-stained fibrillar amyloid in cortex and hippocampus. Lower levels of BACE1 and Aβ oligomers indicated the weakening of amyloid processing pathway. Wang R. et al. [34] showed reduced Congo red stained β-amyloid protein deposits and verified the clearance of Aβ by Huperzine A treatment. 

**Figure 4:**
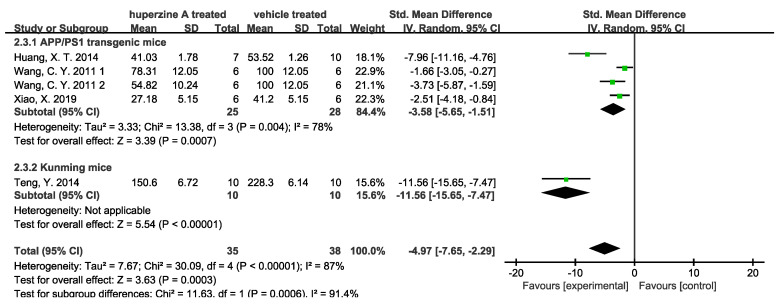
Forest plot for Huperzine A versus vehicle treatment. Outcome: soluble Aβ level measured by ELISA in cortex [26,31,33,37].

**Figure 5:**
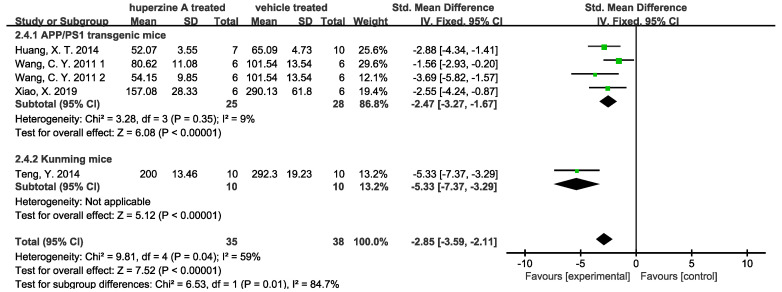
Forest plot for Huperzine A versus vehicle treatment. Outcome: soluble Aβ level measured by ELISA in hippocampus [26,31,33,37].

**Figure 6:**
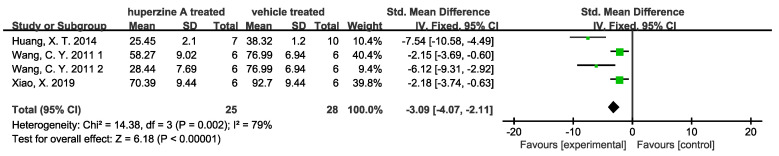
Forest plot for Huperzine A versus vehicle treatment. Outcome: Aβ plaque numbers measured by immunohistochemistry in cortex [26,31,33].

**Figure 7:**
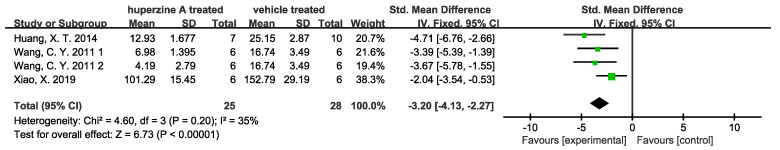
Forest plot for Huperzine A versus vehicle treatment. Outcome: Aβ plaque numbers measured by immunohistochemistry in hippocampus [26,31,33].

Forest plots were used to analyze quantitative results. Four studies used ELISA to quantify Aβ42 level in cortex (Figure 4) and hippocampus (Figure 5). We denoted 1 and 2 for 167 and 500 μg/kg dose of Wang C. Y. et.al. [33] There was a total of 35 mice treated by Huperzine A (25 APP/PS1 transgenic mice and 10 Kunming mice) and 38 treated by a relevant vehicle (28 APP/PS1 transgenic mice and 10 Kunming mice). The soluble Aβ measured by ELISA in both cortex and hippocampus of Huperzine A treated group significantly decreased. In cortex group, the high heterogeneity of APP/PS1 transgenic mice subgroup came from Huang, X.T. et al. [26], when data being excluded could reduce the heterogeneity I^2^ from 78% to 22%. Meanwhile, low heterogeneity (9%) was observed in APP/PS1 transgenic mice subgroup. Three studies used immunohistochemistry to quantify Aβ plaque numbers in cortex (Figure 6) and hippocampus (Figure 7). There was a total of 53 APP/PS1 transgenic mice with 25 treated by Huperzine A and 28 treated by vehicle. The Aβ plaque number in cortex was reduced significantly (*p* < 0.00001) with high heterogeneity (I^2^ = 79%) and in the hippocampus was reduced with low heterogeneity (I^2^ = 35%).

(2) Enhancement of cholinergic activity

**Figure 8:**
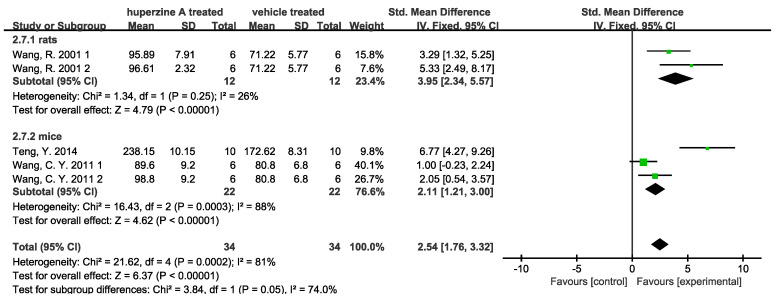
Forest plot for Huperzine A versus vehicle treatment. Outcome: ChAT activity [31,33,34].

**Figure 9:**
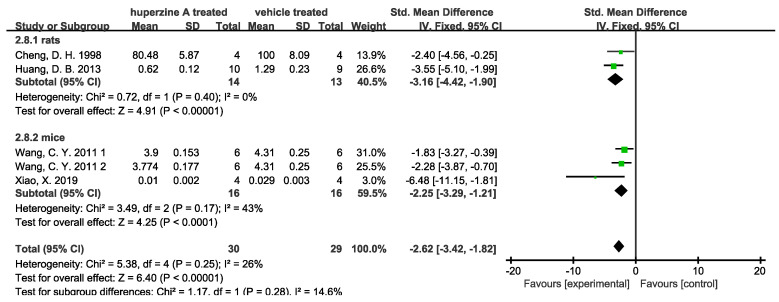
Forest plot for Huperzine A versus vehicle treatment. Outcome: AChE activity [23,25,33,37].

Choline acetyltransferase (CAT/ChAT) was found reduced in the brains of AD patients, while upregulation of cortex and hippocampus ChAT level could prevent the transition process to AD [40]. The currently approved drugs by the FDA to treat AD are AChEIs (donepezil, rivastigmine, galantamine) and an N-methyl-d-aspartate receptor antagonist, memantine. Huperzine A was well known for its activity in inhibiting AChE specifically [41]. Among the included articles, seven studies mentioned the cholinesterase inhibitors (ChEI) efficacy of Huperzine A. Liang Y. Q. et al. [28] injected Aβ peptide into nucleus basalis magnocellularis of rats to test Huperzine A’s effects on cholinergic and monoaminergic dysfunction. They observed greater relief of acetylcholine, dopamine, norepinephrine, and 5-hydroxytryptamine deficit in Aβ injected rats’ cerebral cortex after Huperzine A treatment. Turkseven C. H. et al. [32] compared the cholinesterase inhibition effect of Huperzine A with two other AChEIs (tacrine and E2020). Huperzine A was found to have greater effect in inhibiting AChE in cerebral cortex and hippocampus as well as better at attenuating AF64A-induced memory deficit than those of tacrine and E2020-treated groups. Quantitative results are shown: three studies validated the promotion of ChAT activity (Figure 8) and four validated the inhibition of AChE (Figure 9). Two doses used by Wang R. et al. [34] and Wang C. Y. et al. [33] are displayed in Figure 8, respectively. Promotion of ChAT activity is considerable in the rat subgroup with low heterogeneity (I^2^ = 26%). In the mouse subgroup, the high heterogeneity comes from Teng Y. et al. [31], of which I^2^ would decrease from 88% to 10% if data were removed. The dose 167 μg/kg of Huperzine A used by Wang C. Y. et al. [33] showed no significant promotion on ChAT activity. However, a slight but meaningful rise was observed when the dose increased to 500 μg/kg. AChE activity was inhibited by Huperzine A obviously.

(3) Other effects and mechanisms

Apart from the already mentioned activities, other roles that Huperzine A plays are described by some studies. Teng Y. et al. [31] reported the anti-inflammatory effect and neuron protection effect. Researchers used Huperzine A as a control to test effects of a compound Danshen tablet on Aβ (25-35)-induced memory impairment mice. The mRNA levels and protein levels of IL-6 and TNF-α in hippocampus were greatly reduced by Huperzine A at the dose of 0.4 mg/kg. As for neurotrophins secretion, the BDNF and RACK1 reductions in hippocampus were completely reversed with Huperzine A. The anti-inflammatory effect and neuron protection effect of Huperzine A are comparable to the 0.81 g/kg dose of Danshen.

Huang X. T. et al. [26] reported that Huperzine A significantly inhibited the expression of transferrin receptor 1 (TfR1) and decreased brain iron content in APPswe/PS1dE9 transgenic AD mice. In cultured neurons, Huperzine A reduced transferrin-bound iron uptake. Furthermore, the effect of Huperzine A could be abolished with a high iron diet, which indicated the potential relationship between its neuron protection and reduced brain iron content.

Wang C. Y. et al. [33] reported the anti-oxidation effect and Wnt pathway activation activity of Huperzine A. They treated APP/PS1 mice with a nasal gel containing Huperzine A. Increase in antioxidant enzymes such as CAT and GSH-PX were observed in mouse brains. The phosphorylation levels of GSK3α/β and β-catenin levels were upregulated in vitro and vivo, indicating the activation effect of Huperzine A on the Wnt pathway. Wang Y et al. [36] found that Huperzine A was involved in MAPK pathway as phosphorylated Erk1/2 increased in mice brains with Huperzine A treatment.

**Figure 10:**
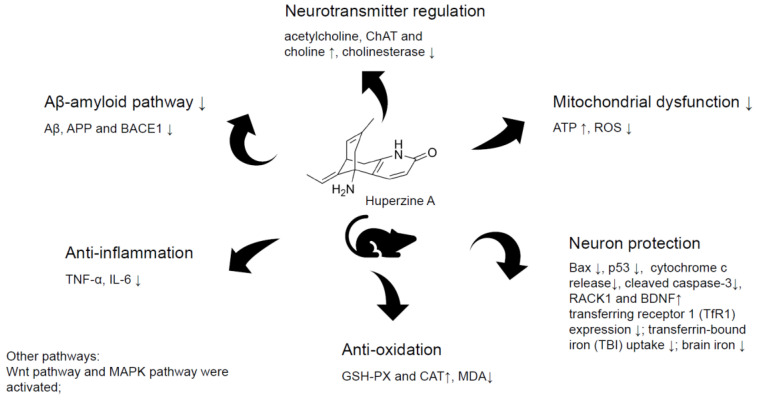
Possible neuroprotective mechanisms of Huperzine A in animal AD models. “↑” means up-regulation, “↓” means down-regulation.

Two studies showed the reduced apoptosis in Huperzine A treatment group. Wang R. et al. [34] reported Huperzine A reduced neuron death, increased BCL protein level, and decreased BAX and p53 protein levels. Xiao X. et al. [37] found that Huperzine A reduced cytochrome c release and cleaved caspase-3 level, indicating the inhibition of apoptosis. They also noticed mitochondrial dysfunction was attenuated significantly by Huperzine A, as ROS levels declined and ATP levels rose. The functions and related possible mechanisms are shown in Figure 10.

### 2.3. Methodological Quality Assessment

We used SYRCLE’s risk of bias tool to assess the methodological quality of the studies included under the instruction of CR Hoojimans. et al. [42]. As Table 3 shows, 1 indicates low risk, 0 indicates unclear risk, -1 indicates high risk. As a result, high scores suggest more reliable methodology and data, and lower scores suggest otherwise more irregular and untrustworthy data. 

The scores range from 2 to 6 with a total score of 10. All studies claimed to provide complete data using such words as “all data/results were present”, so we did not try to find data that might be hidden. Seven studies reported baseline characteristics and six studies reported random housing. Two studies made some effort, limiting the numbers of animals sacrificed, which were classified as low risk in other biases (j). However, only one study (Wang T. et al. [35]) reported sequence generation and allocation concealment. There were three types of bias that could hardly be assessed: performance blinding, random outcome assessment, and detection blinding due to the difficulty to have access to whole protocol of animal experiments in details.

**Table 3:** Characteristics of AD models used in the included studies.

Study	a	b	c	d	e	f	g	h	i	j	Scores
Cheng, D. H. 1998 [23]	0	1	0	0	0	0	0	1	1	0	3
Feng, J. H. 2017 [24]	0	0	0	0	0	0	0	1	1	0	2
Huang, D. B. 2013 [25]	0	0	0	0	0	0	0	1	1	0	2
Huang, X. T. 2014 [26]	0	0	0	0	0	0	0	1	1	0	2
Huang, Z. S. 2009 [27]	0	0	0	0	0	0	0	1	1	1	3
Liang, Y. Q. 2008 [28]	0	1	0	1	0	0	0	1	1	0	4
Nie, H. 2013 [29]	0	0	0	0	0	0	0	1	1	0	2
Rispoli, V. 2013 [30]	0	1	0	1	0	0	0	1	1	0	4
Teng, Y. 2014 [31]	0	1	0	0	0	0	0	1	1	0	3
Turkseven, C. H. 2017 [32]	0	0	0	0	0	0	0	1	1	0	2
Wang, C. Y. 2011 [33]	0	0	0	0	0	0	0	1	1	1	3
Wang, R. 2001 [34]	0	1	0	0	0	0	0	1	1	0	3
Wang, T. 1998 [35]	1	1	1	1	0	0	0	1	1	0	6
Wang, Y. 2012 [36]	0	0	0	1	0	0	0	1	1	0	3
Xiao, X. 2019 [37]	0	0	0	1	0	0	0	1	1	0	3
Xu, Z. W. 2006 [38]	0	1	0	1	0	0	0	1	1	0	4

a: Sequence generation. b: Baseline characteristics. c: Allocation concealment. d: Random housing. e: Performance blinding. f: Random outcome assessment. g: Detection blinding. h: Incomplete outcome data. i: Selective outcome reporting. j: Other bias.

## 3. Discussion

Results from the included studies indicated that Huperzine A could improve memory function, decrease Aβ levels, promote ChAT activity, and inhibit AChE activity. The outcomes of Aβ level and plaque numbers reduction in hippocampus, as well as AChE activity inhibition showed low heterogeneities. Regardless of the well-known inhibitory function of Huperzine A on AChE activity, these results also indicated that Huperzine A’s function in reducing Aβ levels and improving memory may be universal. The noticeable heterogeneities of Morris water maze (Figure 1 and Figure 2) may come from the various models used in those studies. As it is mentioned in Table 2, 16 selected articles used 8 different models. As a result, assigning subgroups based on rats and mice may ignore the bias introduced by the model, though we attempt to balance it by using random effects model. However, this reason cannot well explain all sources of heterogeneity. In the outcome of Aβ plaque numbers measured by immunohistochemistry in cortex (Figure 6), all the animals are APPswe/PS1dE9 transgenic mice but still exhibits high heterogeneity. The likely influencing factors may be differences in dosages, administration routes, treatment durations, and operation protocols. 

However, the therapeutic effect of Huperzine A has not been fully demonstrated clinically. A multicenter trial with 210 individuals showed 200 μg BID of Huperzine A had no benefit in ADAS-Cog at 16 weeks [43]. Clinical trial meta-analyses revealed some improvements in cognitive function during 8 to 16 weeks by Huperzine A treatment but no significant change in the activity of daily living [44,45,46]. Due to the poor methodological quality of the clinical trials, the results were in doubt. N. Ghassab-Abdollahi et.al [46] used the AMSTAR tool to assess the methodological quality of six systematic reviews. They found that Huperzine A had positive effects on activities of daily living, but little evidence in vascular dementia and mild cognitive impairment. Data was so insufficient as two reviews’ results derived only from one random clinical test. Similarly, when we examined the methodological quality of included animal studies, we also noticed the inadequate preparation and data presentation of animal studies, which resulted in untrustworthy and insufficient convincing data. 

It should be noticed that several mechanisms of Huperzine A were proposed, including anti-inflammation, anti-oxidation, and anti-apoptosis, inhibiting the amyloid pathway and attenuating mitochondrial dysfunction. Other pathways were also involved, such as the Wnt and MAPK pathways. It is reported SH-SY5Y exposed to Aβ42 showed decreased ATP level and increasing activity of mitochondrial respiratory chain complex IV with NAD+/NADH. This resulted in inhibition of Sirtuin 2 (SIRT2), a member of the deacetylase family, and led to the decreased level of tubulin acetylase and destruction of microtubule network [47]. Xin Gao, et al. confirmed Huperzine A’s effect in easing mitochondrial dysfunction by showing reduced oxygen free radical accumulation in Aβ treated PC12 cells [18]. Yang, Ling, et al. verified Huperzine A ameliorated ATP reduction and mitochondrial swelling in isolated cortical mitochondria [48]. The interesting effect of Huperzine A on mitochondrial dysfunction may further explain its effects of anti-oxidation and anti-apoptosis [49]. Mechanisms independent of cholinesterase inhibition suggested that exploring mechanisms of Huperzine A is necessary and will be informative for further drug research and development. 

In conclusion, we reviewed 16 animal studies from three separate databases to assess the therapeutic effect of Huperzine A on AD, based on criteria mentioned above. The outcomes showed the definite AChE inhibition and ChAT activity enhancement activity of Huperzine A and suggested its convincing effects in improving memory function, as well as decreasing Aβ levels in cortex and hippocampus. Following this, we collected and presented possible mechanisms suggested by these studies. It is worth noting that negative results may not be published. As a result, the outcomes may be in information bias. The quality of studies included are uneven and most of them lack random and blinding methods. More rigorously designed animal studies with different models and drug administrations are still needed to fully characterize the neuroprotective effect of Huperzine A on AD.

## 4. Materials and Methods

### 4.1. Search Strategy

The publications were retrieved from three independent databases: PubMed, Web of Science, and Google Scholar. We did not limit dates or languages, or try to find unpublished data. The selected time range was from 1986 (the time Huperzine A was discovered) to 2022. Yan Yepiao was the reviewer who assessed the qualifications of the publications by reading abstracts or full texts. The keywords used are as follows: 

“(Huperzine a) AND (Alzheimer’s disease)”

“(Huperzine a) AND (Alzheimer disease)”

### 4.2. Inclusion and Exclusion Criteria

The inclusion criteria were applied as follows:

(1) Types of animals: laboratory animals of any breed, age, sex, or strain were included.
(2) Types of involvement: the study must contain at least a control group and a Huperzine A administration group. The control group should include physiological saline or other solvent control.
(3) Types of results: any results that reflected the effects of Huperzine A on Alzheimer’s disease models.

The exclusion criteria were applied as follows:

(1) No access to the full text.
(2) Reviews, case reports, comments, letters, and clinical trials.
(3) Not testing the effect of Huperzine A on Alzheimer’s disease animal models.

### 4.3. Data Extraction and Quality Assessment

The following information for each study is shown in Table 1. We used RevMan 5.4 (Cochrane Community, London, UK) to perform the meta-analysis. Mean difference with 95% confidence intervals (CI) was used for continuous outcomes. We used standardized mean difference (SMD) to eliminate bias for different units used in different studies. The fixed effects model was firstly used, and if there was high heterogeneity (*p* > 0.05 or I2 > 50%), we will use the random effects model.

## Figures and Tables

**Table 1:** Basic information of included studies.

Article	Animal Data	Administration of Hup A	Methods
Cheng, D. H. 1998 [23]	Sprague-Dawley rats (male, 280–350 g); i.c.v. AF64A 3 nmol/side	dosage: 0.3, 0.5, 0.8 mg/kg/day; ad: i.g.; duration: 3 weeks	behavioral test (radial maze test); biochemical experiments (choline acetyltransferase (ChAT) activity, acetylcholinesterase (AChE) activity, cholinesterase (ChE) activity)
Feng, J. H. 2017 [24]	Sprague-Dawley (SD) rats (male and female, 3–4 months old, 250–280 g); Aβ40 peptide 10 μg injection into left hippocampal CA1 area	dosage: 40.5 μg/kg/day ad: i.g.; duration: 4 weeks	behavioral test (Morris water maze); hematoxylin-eosin staining (neuronal morphology); western blot (Cdk 5 protein); RT-qPCR (*cdk5* mRNA);
Huang, D. B. 2013 [25]	Wistar rats (male, 400–550 g, 10 months old); i.c.v. quinoline acid 150 nmol	dosage: 0.3 mg/kg/day; ad: i.p.; duration: 34 days;	behavioral test (morris water maze); biochemical experiments (acetylcholine and choline level, cholinesterase activity); tissue observation (hematoxylin and eosin staining of hippocampus)
Huang, X. T. 2014 [26]	APPswe/PS1dE9 transgenic mice (2 months old)	dosage: 0.1 mg/kg/day; ad: i.g.; duration: 6 months	Immunohistochemistry (tau plaque); Aβ quantification; western blot (metal transporter with or without iron-responsive element); brain iron measurement
Huang, Z. S. 2009 [27]	senescence-accelerated mouse prone/8 (SAMP8); normal control: senescence-accelerated mouse resistant 1 (SAMR1)	dosage: 0.02 mg/kg/day; ad: i.g.; duration: 50 days;	immunohistochemistry (Aβ expression in the cortex and hippocampus); RT-PCR (mRNA levels of BACE and APP)
Liang, Y. Q. 2008 [28]	Sprague-Dawley rats (male, 250–300 g) 10 μg Aβ injection into nucleus basalis magnocellularis (NBM)	dosage: 012, 0.18 mg/kg; ad: i.g.; duration: 21 days	HPLC (ACh level detection, monoamine level detection); immunohistochemistry (detemine Aβ deposition)
Nie, H. 2013 [29]	APPswe/PS1dE9 mice with C57BL/6J background (male and female, 30–35 g)	dosage: 0.25 mg/kg twice a day; ad: i.g.; duration: 4 weeks	behavioral test (independent activity test, Morris water maze);
Rispoli, V. 2013 [30]	Wistar rats (male, 200–250 g, 3 months old); i.c. excitotoxic AMPA injection into NBM	dosage: 0.5 mg/kg; ad: i.p.; duration: 3 weeks	electroencephalogram (EEG) (theta rhythm); behavioral test (Morris water maze; Object recognition test (ORT))
Teng, Y. 2014 [31]	Kunming mice (male, 8–10 weeks old, 38–42 g) i.c.v. Aβ 25–35 10 μg induced AD model	dosage: 0.4 mg/kg; ad: i.g.; duration: 14 days	behavioral test (Morris water maze); ELISA [Aβ42, (ChAT), IL-6, TNF-α,receptor of activated protein kinase C1 (RACK1) and brain-derived neurotrophic factor (BDNF)]; Brain histopathology (neuronal damage); RT-PCR (β-actin, IL-6, RACK1 and BDNF);
Turkseven, C. H. 2017 [32]	Sprague-Dawley rats (female, 13 weeks old, 180–200 g) 100 mg/kg/day d-galactose i.p.	dosage: 0.1 mg/kg/day; ad: i.p.; duration: 3 weeks	behavioral test (Morris water maze, open field test); electro-biophysical tests (extensor digitorum longus muscle contractile properties); immunohistochemistry (Aβ level); RT-PCR, ΔΔCT-PCR (several miRNA level)
Wang, C. Y. 2011 [33]	APP/PS1 (APPswe/PSEN1dE9) transgenic mice; wild-type C57BL/6 mice (male, 6 months old)	dosage: 167, 500 μg/kg; ad: nasal; duration: 1 months	BrdU Staining (neurogenesis); Evan’s Blue Leakage Assay; AChE, ChAT, GSH-PX, CAT Activity; Immunohistochemistry and Confocal Laser Scanning Microscopy (distribution of Aβ in brain); RT-PCR (GAPDH, APP mRNA level); Sandwich Elisa
Wang, R. 2001 [34]	Sprague-Dawley rats (male, 220–280 g) 800 pmol Aβ 40 i.c.v. at day 5, 8, 12	dosage: 0.1, 0.2 mg/kg/day; ad: i.p.; duration: 12 days	neuron morphology (HE staining); TUNEL staining (detect apoptosis); immunohistochemistry (detect plaque); behavioral test (Morris water maze); measurement of ChAT activity
Wang, T. 1998 [35]	Sprague-Dawley rats (male, 220–270 g) scopolamine 0.15 mg/kg i.p. induced memory impairment	dosage: 0.1, 0.2, 0.3, 0.4, 0.5 mg/kg; ad: p.o.; duration: 30 min before test; dosage: 0.05, 0.1, 0.2, 0.4 mg/kg; ad: i.p.; duration: 30 min before test	radial arm maze task;
Wang, Y. 2012 [36]	APPswe/PS1dE9 transgenic mice (7 months old) high iron diet (2.5% carbonyl iron)	dosage: 0.1 mg/kg; ad: i.g.; duration: 5 months	Golgi staining (observe dendritic spine density); Thioflavin S Staining (assess the deposition of fibrous amyloid); Western blotting; Real-Time PCR Analysis;
Xiao, X. 2019 [37]	APPswe/PS1dE9 transgenic mice (male, 2 months old, 20–30 g)	dosage: 0.1 mg/kg; ad: i.p. duration: 6 months	AChE activity measurement; cell viability; mitochondria activity (ROS level detection, ATP level detection, ABAD immunohistochemistry, cytochrome c release; western blot); measure the level and disposition of Aβ (thioflavin S immunostaining, ELISA quantity);
Xu, Z. W. 2006 [38]	Kunming mice (male, 4 weeks old, 16–25 g) 4 mg/kg scopolamine-induced brain lesion	dosage: 0.1, 0.2, 0.3 mg/kg; ad: i.p.; duration: 30 min before test	behavioral test (step-through passive avoidance test, Y-water maze test)

**Table 2:** Characteristics of AD models used in the included studies.

Model	Mechanism	Main Use	Disadvantages
AF64A induced	A cholinergic neuron-specific neurotoxin and an irreversible inhibitor of choline acetyltransferase	Studying of cholinergic damage as a similar characteristic of AD	Fail to reflect the typical pathological changes of AD senile plaques and NFTs
AMPA induced	Cholinergic neuron toxicity	Simulating cholinergic damage of AD	Fail to reflect the typical pathological changes of AD
quinolinic acid induced	Inducing tau phosphorylation	Simulating tau accumulation of AD	Acute toxicity model cannot simulate the progress of AD.
Aβ induced	Aβ peptide-induced neurotoxicity	Studying of Aβ accumulation and toxicity of AD	A large amount of Aβ accumulates locally at the injection site Instead of spreading to the brain
D-galactose induced	D-galactose induced glucose metabolism disorders and increasing free radical level	Studying mechanism of age-related diseases	The mechanism is unclear and cannot reflect pathological features well
scopolamine induced	Non-selective cholinergic receptor blocker	Simulating cholinergic damage of AD	Lack of the characteristics necessary to study the pathophysiology of AD
APPswe/PS1dE9 transgenic mice	Aβ plaques accumulation	Studying the role of APP and PSEN1 in the development of AD	Lack of neurofibrillary tangles and cost highly
Senescence-accelerated mouse prone/8 (SAMP8)	Naturally occurring rapid aging	Studying mechanism of age-related memory defect	The short life is not suitable for long cycle experiment

## Data Availability

Not applicable.
